# Nevoid melanoma of the vagina: report of one case diagnosed on thin layer cytological preparations

**DOI:** 10.1186/1742-6413-4-14

**Published:** 2007-07-03

**Authors:** Franco Fulciniti, Paolo Antonio Ascierto, Ester Simeone, Patrizia Bove, Simona Losito, Serena Russo, Maria Stella Gallo, Stefano Greggi

**Affiliations:** 1 Section of Cytopathology and Dept. of Pathology, National Cancer Institute, Naples, Italy; 2 Dept of Experimental Oncology, National Cancer Institute, Naples, Italy; 3 Dept of Gynecological Oncology, National Cancer Institute, Naples, Italy

## Abstract

**Background:**

Primary melanoma of the vagina is an extremely rare neoplasm with approximately 250 reported cases in the world literature [1-4]. In its amelanotic variant this lesion may raise several differential diagnostic problems in cytological specimens [5]. In this setting, the usage of thin layer cytopathological techniques (Liquid Based Preparations = LBP) may enhance the diagnostic sensitivity by permitting immunocytochemical study without having to repeat the sampling procedure.

The aim of this paper is to describe the cytomorphological presentation of primary vaginal melanoma on LBP since it has not previously been reported up to now, to our knowledge.

**Case presentation:**

a 79-y-o female complaining of vulvar itching and yellowish vaginal discharge underwent a complete gynaecological evaluation during which a LBP cytological sample was taken from a suspicious whitish mass protruding into the vaginal lumen. A cytopathological diagnosis of amelanotic melanoma was rendered. The mass was radically excised and the patient was treated with α-Interferon.

**Conclusion:**

amelanotic melanoma may be successfully diagnosed on LBP cytological preparations. Thin layer preparations may enhance the diagnostic cytomorphological clues to its diagnosis and may permit an adequate immunocytochemical characterization of the neoplasm.

## Case presentation

A 79-y-o lady reported to the outpatient gynaecological ward of our Institution with a recent history of yellowish vaginal discharge and vulvar itching. She was a non-smoker white Caucasian, gravida 6, para 2, with a past history of bilateral breast carcinoma in 1993 and 1994 (both pT1G_2_N_0_M_0 _ductal carcinomas), respectively treated with quadrantectomy with axillary dissection, radiotherapy and oral tamoxifen. At the time of consultation the patient took oral antidiabetics and anti hypertensive drugs. Her general conditions were satisfactory and her routine blood and urine tests were normal except blood glucose (120 mg/ml).

Visual inspection of the genital tract showed the presence of a gray-white sessile mass of the upper vagina protruding into its lumen. The mass did not consent the visualization of the uterine cervix, was fixed to the lateral vaginal wall and was lined by an eroded oozy mucosa covered by a yellowish slime. External genitalia were atrophic, showing reddened epithelium with focal keratotic areas. A clinical diagnostic hypothesis of vaginal carcinoma was put forward and a LBP cytological sample was taken by gently scraping the surface of the lesion with a soft plastic Cervex brush (Rover Medical Intruments, B.V). The cytological sample was fixed into Preservcyt™ and processed by a Thin Prep 2000™ (Cytyc Italy, Rome). Some small forceps biopsy fragments were also taken, which were immediately fixed in 10% buffered formalin and sent for histopathological examination.

## Cytological and immunocytochemical findings

**Figure 1:**
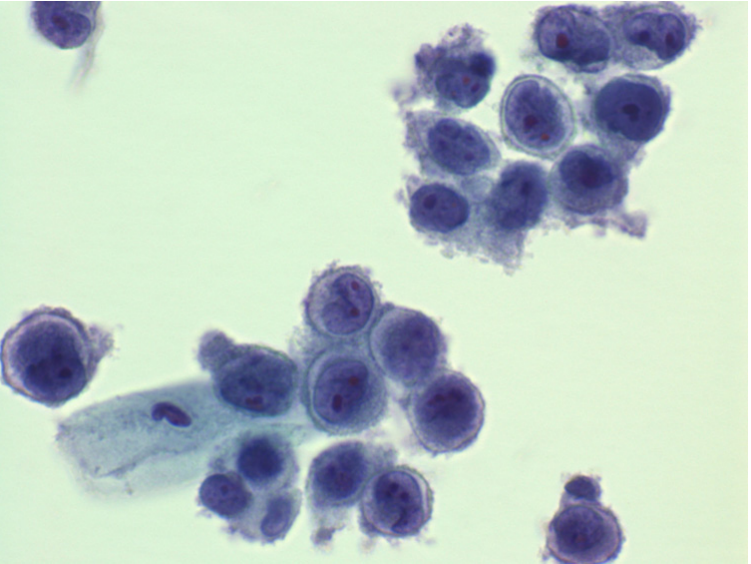
Thin layer preparation, vaginal melanoma. Note the dispersed proliferation of highly atypical medium sized cells with ovoid or kidney-shaped, nucleolated nuclei. The neoplastic cells display a scarce to moderate amount of greyish granular cytoplasm with peripheral condensation and cytoplasmic projections suggestive of hyperplastic microvilli (Papanicolaou stain, 600×, original magnification).

Cytopathological examination of Papanicolaou stained thin layer preparations showed a rather homogeneous population of dispersed neoplastic cells of medium to large size in a clean background. The neoplastic elements outnumbered the normal squamous cells of the vaginal mucosa and displayed generally single, round nuclei with kidney-shaped or irregularly cleft nuclear membrane. The nuclear chromatin was densely granular and one to three prominent nucleoli of irregular shape were present per nucleus. Cytoplasm ranged from scarce to moderate: it was greyish to greenish and had a rather dense appearance with a perinuclear clear halo and an area of ring-like condensation in the vicinity of the cellular membrane; a minority of the cells showed a finely vacuolated cytoplasm. Cytoplasmic borders displayed irregular surface evoking hyperplastic microvilli (Fig. 1).

**Figure 2:**
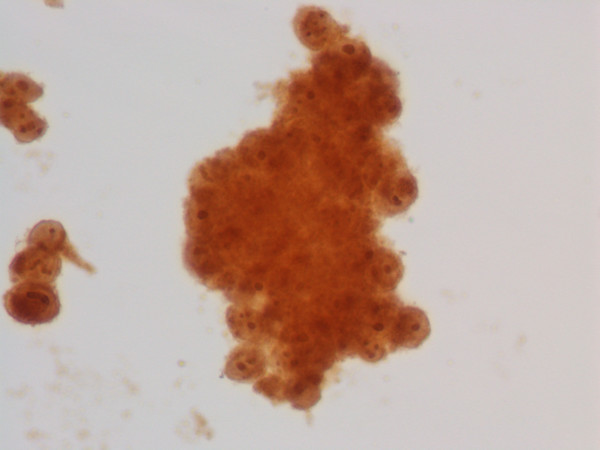
Thin layer preparation, vaginal melanoma: immunocytochemical stain for HMB 45 showing diffuse cytoplasmic positivity in neoplastic cells.

**Figure 3:**
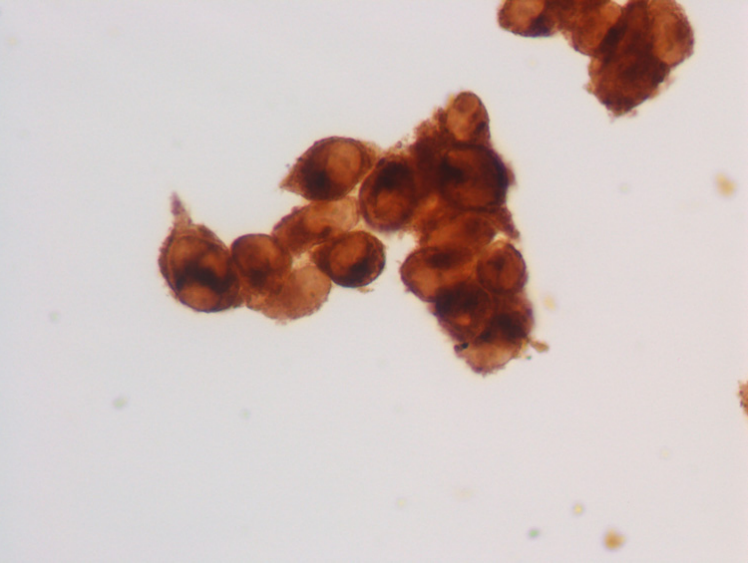
Thin layer preparation, vaginal melanoma: immunocytochemical stain for S-100 protein showing diffuse cytoplasmic positivity in neoplastic cells. Figs. 2 and 3: immunoperoxidase, 400×, original magnification.

Due to the peculiar cytomorphology of the neoplastic cells and to the absence of "tumour diathesis", the clinical hypothesis of vaginal carcinoma was discarded in favour of a possible melanoma diagnosis. New LBPs were obtained from the original sample that were fixed in 95% ethanol and processed for immunocytochemistry for Vimentin (clone V9, diluted 1:80), S-100 (polyclonal antibody, 1:250), pan-cytokeratin (clone 6 F11, 1:80) and HMB 45(clone HMB 45, !:80) by using commercially available antibodies (Novocastra, U.K.) and an automated immunostainer (Dako Autostainer, Dako Italy, Milan). Positive controls were represented by small histopathological sections of known reactivity that were stained in the same run. The neoplastic cells showed a diffuse cytoplasmic positivity for Vimentin, S-100 protein and HMB 45, while pan-cytokeratin staining was negative (Figs. 2 and 3). A cytopathological diagnosis of nevoid (amelanotic) melanoma was made.

## Histopathological findings

The cytopathological diagnosis was confirmed on the biopsy fragments on which a diagnosis of infiltrating epithelioid cell amelanotic melanoma was made.

Magnetic Resonance Imaging demonstrated a 5 × 4 × 4 hyperintense mass in T1, located in the upper left third of vagina infiltrating the vaginal wall, though being apparently confined to it. No evidence of lymphadenopathy or of distant metastases was found.

The patient underwent total abdominal bilateral hystero-annessectomy with vaginectomy and left inguino-femoral lymphadenectomy.

**Figure 4:**
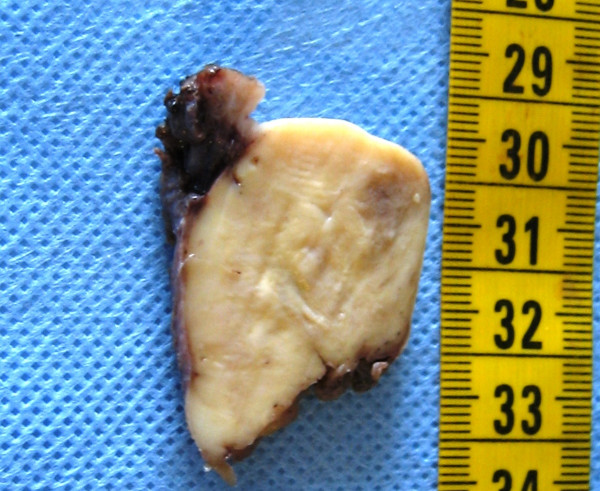
Vaginal melanoma, cross-section of surgical sample. A 5 × 4 × 4 cm whitish solid mass can be observed lined by partly ulcerated vaginal mucosa. The left cervical fornix is visible in the upper center of the picture.

**Figure 5:**
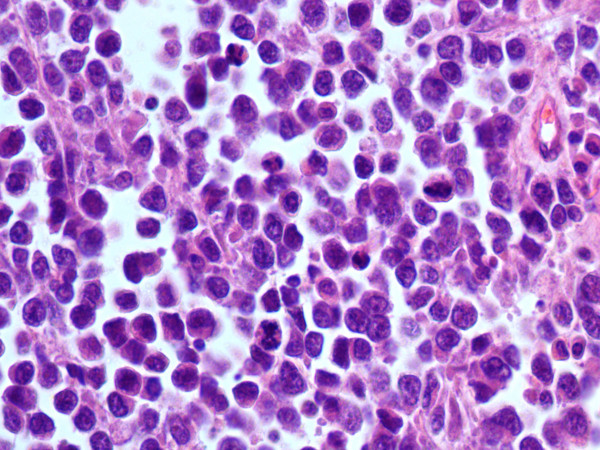
Vaginal melanoma: histopathological section showing an infiltrating dispersed cell population with alveolar pattern of growth (Hematoxylin and Eosin, 250×, original magnification).

**Figure 6:**
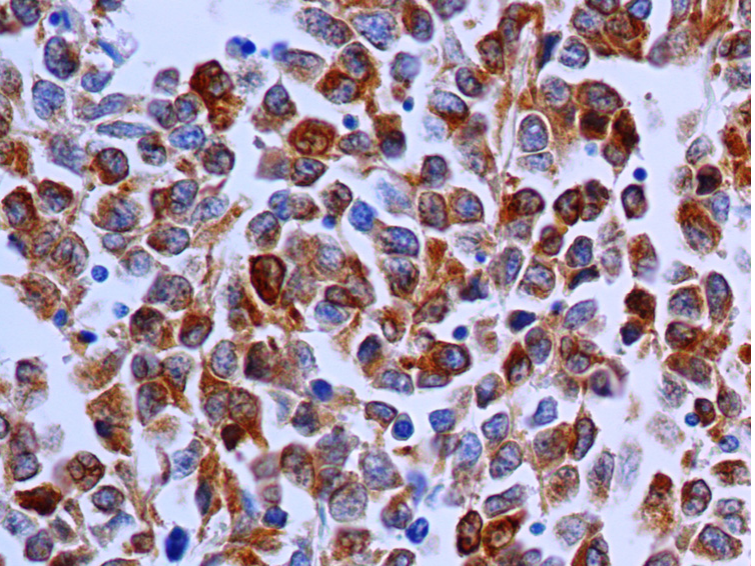
Immunocytochemical staining for MART-1 demonstrating diffuse cytoplasmic positivity of the neoplastic cells (Immunoperoxidase, 250×).

A 5 × 4 × 4 cm sessile mass was found protruding in the lumen of upper third of vagina, near the left lateral fornix. The mass had a solid, rather homogeneous grey to white cut surface and was lined by a greyish pseudomembrane made up of fibrin and remains of ulcerated squamous epithelium (Fig. 4). The histopathological picture showed a solid, infiltrating epithelioid cell proliferation with alveolar pattern, lacking an obvious intraepithelial component apparently due to the diffuse surface ulceration. The neoplastic cells displayed intensely hyperchromatic cleft or kidney-shaped nuclei with prominent nucleoli and dense, deeply eosinophilic cytoplasms. Immunocytochemical stains for Vimentin, S-100 protein, HMB 45 and MART-1 (Dako M 7196, 1:50) were positive, while pan-cytokeratin stain was negative. A diagnosis of ulcerated amelanotic melanoma with a prevalent epithelioid cell component was made (Figs. 5 and 6). The neoplasm was 2.3 cm-thick according to Breslow and stage IV according to Chung.

All the 13 lymphnodes isolated from the lymphadenectomy sample were free of disease as well as the lower surgical margin. The uterus and annexa were pathologically irrelevant.

The patient was put on α-interferon therapy (3,000,000 units subcutaneously three times per week) and is well at the time of writing, three months after the operation.

## Differential diagnosis and discussion

The cytological finding of a dispersed proliferation of highly atypical cells of medium size in a gynaecological specimen, in the absence of necrosis, readily evoked the possibility of melanoma. Although rare, vaginal melanoma has in fact already been diagnosed on traditional cytological gynecological smears [5-9] but, to our knowledge, this is the first case diagnosed on a routine LBP exfoliative sample. Melanoma has, however, previously been described also on LBP samples prepared by fine-needle aspirates [10,11]. These reports underline some minor differences consisting in a reduction of the cellular clusters, coarser nuclear chromatin and a statistically significant reduction in the number of intranuclear cytoplasmic inclusions in LBP as compared to traditional smears. Our findings are in general agreement with the observed ones on LBP of fine needle aspirates. Moreover, the absence of cellular necrosis (which is not frequent but may well be seen in traditional smears of melanoma), was notable in our case.

Other differential diagnoses which were initially entertained in consideration of this presentation and of the age of the patient were high grade stromal sarcoma of the endometrium, high grade invasive cervical carcinoma or endometrial adenocarcinoma and hemato-lymphoid neoplasms. To our knowledge, no cases of endometrial stromal sarcoma have been described on thin layer samples. Becker and Wong [12] described the peculiar cytological presentation of stromal endometrial sarcoma on traditional smears, underlining the frequent "tumour diathesis" associated to single neoplastic cells of small size, frequently showing tapered or elongated "comet" shapes. These cellular elements may be seen admixed to normal endometrial glandular cells [13] or to hyaline metachromatic extracellular material in fine needle aspiration samples [14]. The complete absence of "tumor diathesis" and of a biphasic cell population strongly weighed against stromal endometrial sarcoma, while the prevalence of medium size dispersed cells which were negative on pan-cytokeratin staining excluded poorly differentiated squamous carcinoma and adenocarcinoma.

The possibility of a hemato-lymphoid neoplasms was not considered in our case, but, it should be at least evaluated in the differential diagnosis of all types of dispersed cell populations in a gynaecological specimen. Most hematological malignancies may primarily or secondarily involve the genital tract: in our case lymphoblastic [15] or myeloid [16] leukemia could have been excluded based on the cell size and nucleo/cytoplasmic fetures, while high grade non-Hodgkin lymphoma [17] and anaplastic myeloma [18] could have been the only possible differential due to the nucleolar prominence and large cell size. Some nucleo-cytoplasmic features of the cell population in our case, however, would have strongly dictated against these two entities: the presence of peripheral ring-like cytoplasmic condensations, of irregular cytoplasmic borders evoking hyperplastic microvilli and the extremely lobulated and convoluted shape of the nuclear membrane: this latter seemed excessive even for a high grade lymphoma of convoluted cell type.

The adoption of a LBP cytopathological sample permitted us in this case to complete all the immunocytochemical tests by using the same original sample, without having to recall the patient for further refinement of the cytopathological diagnosis.

In conclusion this report, besides to showing the cytological features of a rare lesion of the genital tract on thin layer preparations, demonstrates how the higher technical costs of LBP methodologies may be strongly outbalanced by the clinical advantages obtained by the adoption of these techniques in gynaecological oncology. In the present case LBP permitted the obtain multiple preparations which could be used for ancillary techniques saving critical turn-around diagnostic time and permitting timely surgical treatment. In these concern, the writers strongly encourage the diffusion of thin layer cytopathological techniques in gynaecological oncology.
